# Validity of Valor Inertial Measurement Unit for Upper and Lower Extremity Joint Angles

**DOI:** 10.3390/s24175833

**Published:** 2024-09-08

**Authors:** Jacob Smith, Dhyey Parikh, Vincent Tate, Safeer Farrukh Siddicky, Hao-Yuan Hsiao

**Affiliations:** 1 Department of Kinesiology and Health Education, The University of Texas at Austin, Austin, TX 78712, USA; jacobmsmith@utexas.edu (J.S.); safeer.siddicky@austin.utexas.edu (S.F.S.); 2 Valor Biomechanics, Austin, TX 78703, USA; ddparikh@valorbiomechanics.com (D.P.);

**Keywords:** biomechanics, inertial measurement unit, motion capture, validation, wearable sensors

## Abstract

Inertial measurement units (IMU) are increasingly utilized to capture biomechanical measures such as joint kinematics outside traditional biomechanics laboratories. These wearable sensors have been proven to help clinicians and engineers monitor rehabilitation progress, improve prosthesis development, and record human performance in a variety of settings. The Valor IMU aims to offer a portable motion capture alternative to provide reliable and accurate joint kinematics when compared to industry gold standard optical motion capture cameras. However, IMUs can have disturbances in their measurements caused by magnetic fields, drift, and inappropriate calibration routines. Therefore, the purpose of this investigation is to validate the joint angles captured by the Valor IMU in comparison to an optical motion capture system across a variety of movements. Our findings showed mean absolute differences between Valor IMU and Vicon motion capture across all subjects’ joint angles. The tasks ranged from 1.81 degrees to 17.46 degrees, the root mean squared errors ranged from 1.89 degrees to 16.62 degrees, and interclass correlation coefficient agreements ranged from 0.57 to 0.99. The results in the current paper further promote the usage of the IMU system outside traditional biomechanical laboratories. Future examinations of this IMU should include smaller, modular IMUs with non-slip Velcro bands and further validation regarding transverse plane joint kinematics such as joint internal/external rotations.

## 1. Introduction

Biomechanical analysis of human movement has increasingly gained popularity among clinicians for examining healthy and clinical populations in a wide variety of fields including academia, athletics, and private sector industries. Obtaining whole-body kinematic measurements traditionally requires optical motion capture cameras, retroreflective markers, force plates, and powerful data processing machines to record and analyze human movement. This setup is currently the gold standard [1,2] but is generally restricted to laboratory environments that face the following issues: high cost, lack of portability, a time-consuming setup, and an extensive post-processing time [3]. Previous literature has reported that human kinematics differ between laboratory and field environments [4] and moving out of laboratory environments is suggested to capture real-world movements [5,6]. Therefore, utilizing approaches that allow for accurate field kinematic analysis is warranted.

A popular alternative to traditional motion capture is a wearable inertial measurement unit (IMU). IMUs are commonly used to examine biomechanical performance outside a traditional laboratory environment. These wearable devices allow portability for in-field assessments including upper and lower extremity movements, posture, and trunk movements [7,8,9]. In addition, these systems provide time-friendly analysis and are a cost-efficient substitute when compared to traditional optical motion capture systems. In fact, rehabilitation settings have used commercial IMUs for motion and gait analysis [10], while also providing clinicians with long battery life for analyzing human motion [11], which is an important factor to consider when choosing an IMU system [12]. However, IMU systems can have drawbacks such as sensor drift caused by magnetic disturbances [13], and inadequate calibration routines [14,15], which may hinder the reliability and accuracy of an IMU. Thus, the validation of IMU metrics in assessing human movement must be examined. 

The Valor (Valor Biomechanics, Austin, TX, USA) IMU system aims to offer a low-cost and time-friendly alternative to traditional optical motion capture (Vicon, Oxford, UK) while achieving accurate kinematic analysis. This system has produced a novel software (v2.10) component, paired with the IMUs, that provides a more efficient and actionable way to utilize motion capture data for assessing mobility and performance beyond just a measurement tool. Furthermore, the system presents a movement-agnostic approach that covers exercises that assess everything from range of motion to skill-based techniques, such as change of direction on the field, to help correlate how training and game performance can have a deeper interplay with each other. It is therefore important to validate the capability of the Valor system by tracking joint angles, as these form the foundation of the paired software, which analyzes and breaks down this data into a more actionable format. Many similar commercial inertial measurement systems have also undergone validation, setting a precedent for the need to determine how well they can perform when compared to high-end optical motion capture including the Xsens IMU (Xsens, Enschede, the Netherlands), Perception Neuron (Noitom, Miami, FL, USA), and Noraxon IMU (Noraxon USA, Scottsdale, AZ, USA) systems [16,17,18]. However, most of these IMU-based technologies on the market, including popular systems such as Movella (Xsens, Enschede, the Netherlands), do not currently provide a holistic approach to collecting, analyzing, and personalizing motion capture data for enhanced industry-specific use cases such as sports, physical therapy, health workers, and many more. Additionally, with the overall improvement and use of IMU technology, many at-home or in-lab IMU systems have also been validated and systematically reviewed to determine their potential to track joint angles in human activity [19]. Therefore, the purpose of this study is to validate upper and lower extremity joint angles obtained by the all-in-one Valor IMU and software. We hypothesized that when compared to a Vicon motion capture system, the Valor IMU would achieve a joint angle mean root-mean-squared-error (RMSE) of 10 degrees or less and interclass correlation coefficient (ICC3k) reliability of 0.90 across a range of dynamic movements including single leg squat, bilateral squat, vertical jumping, shoulder flexion/extension and abduction/adduction, and deadlifting.

## 2. Materials and Methods

### 2.1. Subject Demographics

Twenty healthy young adults (8 male; 12 female) between 18 and 35 years of age (22.46 ± 3.61) who had no diagnosed musculoskeletal injury in the past six months and had at least one year of deadlifting experience participated in this study. Each subject’s height (1.70 ± 0.10 m), weight (80.07 ± 26.61 kg), and self-reported lift weight, with one repetition max (1RM) for conventional deadlifts, were obtained (46.16 ± 12.92 kg). Additionally, subjects reported their years of experience deadlifting (4.53 ± 3.56). The participant’s dominant leg was determined by the leg used to kick a soccer ball for a maximum distance by self-report. All subjects reported their right lower limb as the dominant limb. All subjects read and signed an informed consent form and a physical activity readiness questionnaire 2023 [20] for a one-time visitation. This study was approved by the University of Texas and the Austin Institutional Review Board.

### 2.2. Optical Motion Capture System Instrumentation 

Kinematic data were collected using a twelve-camera Vicon motion capture analysis system (Vicon, Oxford Metrics, UK) at 120 Hz. Kinetic data were collected at 1200 Hz using two force plates (Bertec, OH, USA) to capture ground reaction forces beneath each foot during all tasks. Force plate data were low-pass fourth-order Butterworth filtered, with a cutoff frequency of 20 Hz, and were normalized to body weight in Visual3D (C-Motion Inc., Germantown, MD, USA). Marker trajectories were filtered using fourth-order Butterworth low-pass, filtered at 6 Hz in Visual3D (C-Motion Inc., Germantown, MD, USA). Reflective marker placement corresponded to the Vicon Full-Body Plug-In-Gait Model [21].

### 2.3. Valor IMU Instrumentation

**Figure 1:**
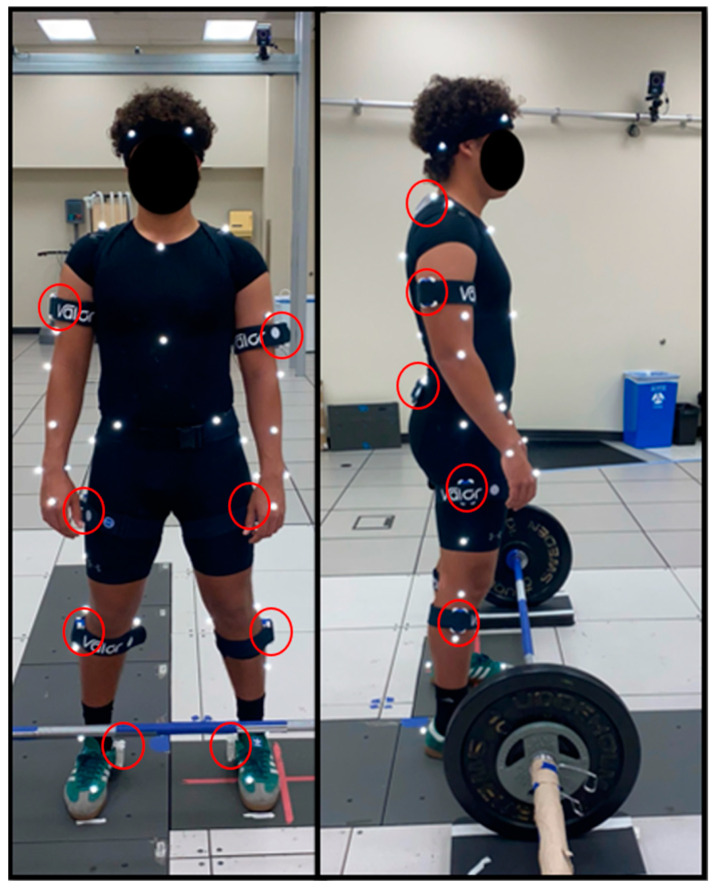
Representative illustration of Valor inertial measurement unit placement. Ten Valor Velcro straps are used to secure each Valor IMU to the subjects’ ankles, shanks, thighs, waist, trunk, and upper arms, as seen marked by the red circles.

IMU data was collected at 30 Hz using 10 wearable Valor IMU sensors (Valor Biomechanics) to capture bilateral ankle, knee, hip, and shoulder joint angles during all tasks, as seen in Figure 1. Additionally, the Valor IMU system has a two-step calibration process before capture: manual calibration of the sensors themselves followed by a pose calibration once the sensors have been properly placed onto the participant. The manual calibration requires turning each sensor on, rotating them individually in a 360-degree fashion until the commercially available software paired with the Valor IMU notifies that the sensor has been well calibrated to the environment, and then placing them flat on a level surface for approximately 3 s. The second calibration step is performed after each sensor has been placed onto the participant and is based on two poses: the inverted neutral pose (a pose that follows the typical neutral pose but with arms extended above the head with palms facing toward each other) and the neutral pose, in that respective order. Each pose is required to be held by the participant for 5 s until the paired software notifies them that each pose has undergone calibration.

All data from the Valor IMUs was obtained and processed using accompanying commercial software from Valor Biomechanics. The data collected in real-time was raw IMU-fused orientation data represented as quaternions and then sent to an online cloud-based database where it was converted into joint kinematics data. The data was further post-processed and up-sampled at 60 Hz using a moving average filter and interpolation. All processing was performed in a closed system and the final data was presented via JSON files. 

### 2.4. Procedure

Participants were provided tight-fitting clothing to change into for data collection. Subject anthropometric data including height, weight, bilateral ankle, knee, shoulder offset, elbow, wrist, and hand thickness were obtained, along with bilateral leg length. Retroreflective spherical markers were attached to each subject following the Vicon Nexus Full-Body Plug-In-Gait marker set. After reflective marker placement, 10 Valor IMUs were attached to the upper back (trunk), pelvis, thighs, calves, feet via shoe clips, and triceps, as seen in Figure 1. Specifications and guidance were provided by Valor Biomechanics on best practices and how to attach each IMU using the label information on each IMU and ensuring that they were placed as close as possible to the middle of the limb segments they were to be placed on. The labels specifically provided information regarding which limb the IMU was supposed to be placed on (e.g., Left Thigh or Right Tricep). Additionally, it was recommended to ensure that each IMU had the labels facing in an upward direction which was signified by arrows on the label of each IMU. Following the placement of markers and IMUs, the two-pose Valor IMU static calibration was performed to map each IMU to a specified joint center in a biomechanically rigged model. Participants then performed separate trials that consisted of the following: one trial of four left shoulder abduction/adduction repetitions, one trial of four right shoulder abduction/adduction repetitions, one trial of four dominant limb single leg squat repetitions, one trial of four non-dominant single leg squat repetitions, one trial of four body weight squat repetitions, four separate trials of a single repetition of a vertical jump with hands on hips, and two separate trials of deadlifting. The first deadlifting trial consisted of one set of five conventional deadlifting repetitions employing a mixed grip style, with the second trial employing a double-overhand grip style. All trials were performed in order and at a self-selected pace by the participant.

### 2.5. Data Analysis

For each joint angle-movement combination (e.g., Left Ankle Angle-Bilateral Squat), the peak-to-peak joint angular excursion during each repetition was quantified and then averaged across repetitions. Next, the difference between the values from Valor and the motion capture system was calculated. The mean square error was calculated from the peak subsets of both systems, then the square root was taken. After this was calculated for all subjects in each joint angle-movement combination, the final result was averaged across participants, yielding one RMSE value for each joint angle-movement combination. This was calculated similarly to absolute difference, where all peak values from both systems for a given joint angle-movement combination were placed into two arrays, then the ICC3K was calculated in Python 3.12 (Python Software Foundation). The data array is of the same n × m × s length as the absolute difference. The breakdown of the data array, as aforementioned, is as follows: n is the number of repetitions or trials within each movement dataset, m is the number of movement datasets for each participant, and s is the number of participants.

### 2.6. Statistical Analysis

The mean absolute difference, RMSE, and ICC3k with 95% confidence intervals were calculated for all joint angles when comparing the Valor IMU to the Vicon optical motion capture. Additionally, a paired *t*-test against one sample, or a one-sample *t*-test, was performed to determine if the statistical significance of the mean difference between the Valor IMU and Vicon systems was less than 10 degrees, as hypothesized. All statistical analysis was performed in Python 3.12 utilizing NumPy and Pandas manipulation along with SciPy, scikit-learn, and Pingouin.

## 3. Results

### 3.1. Root-Mean-Squared-Error

**Figure 2:**
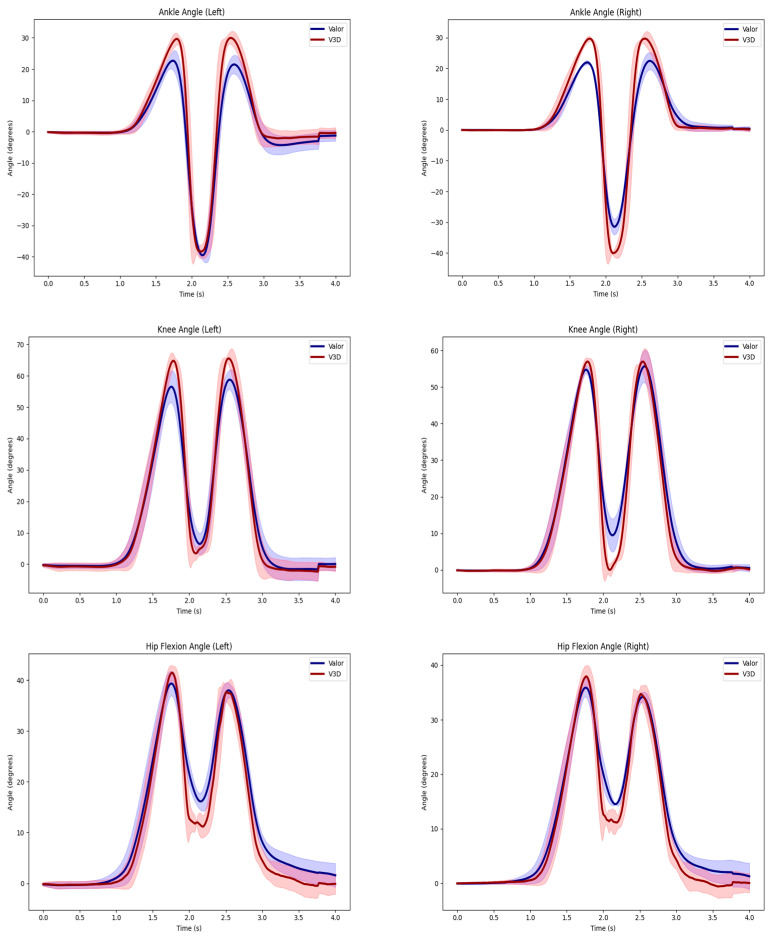
Left and right ankle, knee, and hip joint angles are represented by the average curves between Vicon motion capture (V3D), shown in red, and the Valor inertial measurement unit (Valor), shown in blue. Data from a representative subject performing the vertical jump task.

Valor-derived RMSE joint angle values across all subjects and tasks reported a range from 1.89 degrees to 16.62 degrees relative to joint angles obtained by Vicon (Table 1 and Table 2). The right ankle angle reported the lowest mean RMSE value of 3.3 degrees during the left limb single-leg squat while the left hip angle reported the highest mean RMSE value of 13.22 degrees during the deadlift in the lower extremity (Table 1) across all subjects and tasks. All lower extremity joint angle mean RMSE values were reported below 10 degrees (Table 1) (Figure 2). 

**Figure 3:**
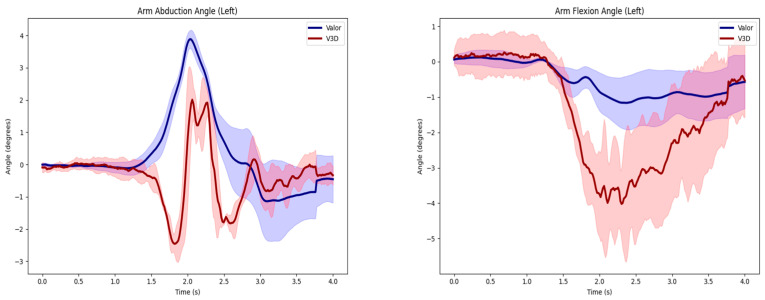

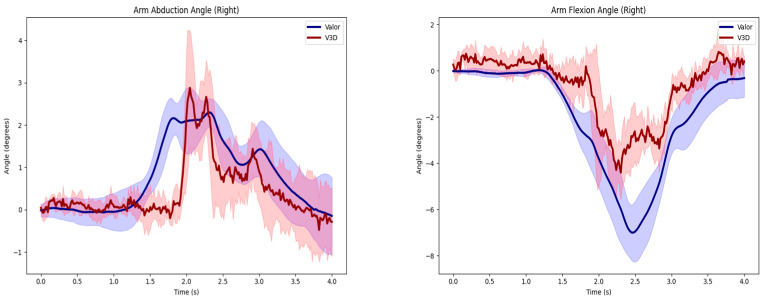
Left and right shoulder joint abduction and flexion angles are represented by the average curves between Vicon motion capture (V3D) shown in red, and the Valor inertial measurement unit (Valor), shown in blue. Data from a representative subject performing the vertical jump task.

Regarding shoulder joint angles, the right shoulder abduction angle reported the lowest mean RMSE value of 1.89 degrees during the left shoulder abduction, while the right shoulder abduction angle reported the highest mean RMSE value of 16.62 degrees during the deadlift (Table 2). However, the mean RMSE value observed in left shoulder flexion was reported to be above 10 degrees (Table 2), while the mean RMSE values in the left and right shoulder abduction and right shoulder flexion movements were below 10 degrees (Figure 3).

### 3.2. Interclass Correlation Coefficient

When comparing Valor-derived joint angles to Vicon-derived joint angles, ICC3k values (Table 3 and Table 4) across all subjects and tasks ranged from the lowest mean ICC3k value of 0.57 reported in the left shoulder flexion angle, to the highest mean ICC3k value of 0.99 reported in right abduction/adduction. In the lower extremity, a majority (26/30) of the calculated ICC3k values for lower-body joints fall in the “excellent consistency” category (0.9–1.0) or “good consistency” category (0.75–0.9). In the shoulder joints, five of the calculated ICC3k values were classified in the “excellent consistency” or “good consistency” categories [22].

### 3.3. Paired T-Tests

By utilizing the mean absolute differences found between the Valor IMU and Vicon systems (see Supplementary Data), simple paired *t*-tests were performed for each joint angle to determine if the difference was statistically significant, in comparison to being less than 10 degrees (Table 5). Across all joint angles, the extremely small *p*-values obtained demonstrated strong evidence, with statistical significance, that the differences between the Valor IMU system, on average, were within or less than 10 degrees of the Vicon system’s measurements. 

## 4. Discussion

The purpose of this investigation was to examine the validity of the Valor IMU-derived joint angles of the shoulder, hip, knee, and ankle when compared to traditional optical motion capture. Our initial hypothesis was partially supported regarding the Valor IMU obtaining RMSE values of 10 degrees or less for all joint angles evaluated in the present study.

The Valor IMU comparison revealed that the largest RMSE values in the lower extremity were reported in the left and right hip joint angle during deadlifting (Table 1), while the largest RMSE in the shoulder joint was reportedly the left and right shoulder flexion during deadlifting (Table 2). Valor IMUs achieved an RMSE value of fewer than 10 degrees in 83% of tasks to joint angle comparisons in the lower extremity joints and less than 10 degrees in 71% of tasks to joint angle comparisons in the shoulder joint angles. However, it should be noted that data were not made available for left and right shoulder abduction during the deadlift due to processing issues. The higher reported RMSE values can also be explained by magnetic disturbances caused by the metal barbell used in this study, as IMU accuracy has been shown to decrease when encountering metal objects [23,24]. This is especially true during the full extension of a deadlift where the barbell is closest to the pelvis for an extended period of time relative to the ankle and knee joint. Furthermore, it was observed that some subjects grazed the barbell along the anterior portion of the thigh segment in lifting and lowering the barbell to complete the deadlifting task which could have shifted the thigh IMU Velcro strap as repetitions progressed. Regarding shoulder joint angles present in this study, we found that RMSE values exceeded 10 degrees. However, a previous study reported RMSE values of an IMU-captured shoulder joint angle of up to 12.1 degrees [25] which is within the margin of the shoulder flexion RMSE results in the current study. While this did not support our hypothesis of achieving below 10 degrees of error, future research and development should be considered to optimize IMU capture capabilities in the upper extremity. 

Furthermore, most of the observed ICC3k values reported either “good consistency” or “excellent consistency” when comparing joint angles captured by the Valor IMU to the Vicon. More specifically, we observed that 56% of lower extremity task to joint angle comparisons were of “good consistency” (Table 3) and roughly 43% of these comparisons were of “excellent consistency” (Table 4). These findings reveal valuable insight into the IMU’s accuracy against an industry gold standard of optical motion capture as previous studies have utilized ICC to validate joint kinematic capturing capabilities [26,27]. Regarding shoulder joint angle ICC3k observations between the two systems, 30% of the task to joint angle comparisons were classified as “good consistency”, 20% of comparisons were labeled as “excellent consistency”, and the remainder were categorized between poor to moderate consistency. One explanation of these findings could be the design of the trunk IMU Velcro band, which rests between the scapulae. This positioning means that shoulder flexion or abduction could influence the trunk IMU to shift during these movements. While we also acknowledge ferromagnetic interference could have played a pivotal role in our findings, further examinations need to be performed to assess the optimal attachment site for the trunk IMU.

To further strengthen the conclusion of partial support that the Valor IMU system is within 10 degrees or less of the Vicon system, the paired *t*-tests across all results indicated that the differences between each system were significant, thus rejecting the null hypothesis that the joint angle differences between both systems would be greater than 10 degrees. These findings, paired with the RMSE and ICC findings, demonstrate that there is variability between the joint angles derived from the Valor IMU and Vicon systems. Overall, most joint angles were within the hypothesized difference of 10 degrees but depending on the exercise and inherent limitations discussed below with the Valor system, the difference could exceed this threshold. 

Another important point of discussion for this study is how the Valor IMU system compares to other IMU systems seen within the market and in the industry for human motion capture applications. Some of the most utilized IMU setups include the Perception Neuron platform (Miami, FL, USA) and Xsens IMU system (Enschede, The Netherlands) which have undergone similar validation studies against optical motion capture systems. These studies report utilizing similar measures of comparing accuracy between wearable motion capture and optical systems. Studies validating the Neuron or Xsens IMU systems demonstrated RMSE values like the Valor IMU, ranging between 4.9 and 10.5 degrees or between 6 and 18 degrees, respectively, across multiple joint angles [16,17]. Similar results have been reported between different correlation coefficient measures within these studies (ICC vs. coefficient of multiple correlation: CMC vs. cross-correlation: XCORR) for such systems ranging from 0.26 to 0.96 XCORR values for Xsens and from 0.78 to 0.96 CMC values for Neuron across multiple joint angles [16,17]. This comparison validates the potential of the Valor IMU for tracking complex human movements outside of the laboratory environment and suggests it has adequate sensitivity to measure changes in joint angles for identifying deviations in movement patterns. 

This study is not without limitations. A commonly known concern is surrounding drift with IMU technology, even with a 9-axis sensor fusion and calibration between an accelerometer, magnetometer, and gyroscope sensors. Other limitations are related to IMU placement on subjects, including the shifting of sensors due to skin and clothing artifact displacement during tasks, which were most present for the pelvis, thigh, and ankle IMUs. Additionally, while anthropometric measurements were recorded for the placement of retroreflective markers, the IMUs did not follow the same strict protocol resulting in possible differences in segment tracking of the Valor IMU system. Future examinations of this IMU should include smaller modular IMUs with non-slip Velcro bands, and further validation surrounding transverse plane joint kinematics, such as joint internal/external rotations. Another limitation present in IMU technology and within the Valor IMU, as mentioned before, is magnetic disturbances caused by metallic objects. Possible solutions to resolve this issue involve custom algorithms or filters such as extended or unscented Kalman filters developed to threshold the fusion between the separate components of IMUs. This requires an in-depth understanding of the intended use of magnetometer readings within an IMU and how to limit the reliance on this data when paired with gyroscope information, to reduce picking up magnetic disturbances. 

## 5. Conclusions

There is an increasing demand for IMU systems to conduct biomechanical analysis of human movement. This study assesses the accuracy of the commercially available Valor IMU for capturing ankle, knee, hip, and shoulder joint angles across various movements. With the results obtained from this study, it is determined that the Valor IMU system maintains the potential to perform tracking of complex human movements outside a laboratory setting. The potential applications for this range from assessing athlete mobility to at-home screening of functional needs, which is generally utilized by physical therapy patients. However, limitations include the use of separate bands or placement mechanisms for each IMU versus a holistic garment that presents possible fluctuations in the accuracy of the joint range of motion collected across the body, as mentioned in the discussion. This is likely due to direct effects on the pose calibration routine of the Valor system, where sensor placement may cause potential underestimation or overestimation of joint range of motion if not directly placed on what are true anatomical joint and segment landmarks. Additionally, while the full wireless capability of the Valor IMU presents an easier way of rapidly tracking various human activities, interference during data transmission does pose further limitations when it comes to utilizing such technology for more rapid movements found in sport-specific skill techniques. Future research and development should focus on the validity of movements occurring in the transverse plane, along with mitigating possible sources of interference to the IMUs caused due to wireless communication via Bluetooth or Wi-Fi, and magnetic field fluctuations when near metallic objects. 

## Figures and Tables

**Table 1:** Valor IMU vs. Vicon RMSE in Lower Extremity Joint Angles. The table shows the upper extremity joint angle mean root-mean-squared-error (RMSE) observations when comparing Valor-derived joint angles to Vicon-derived joint angles across all subjects and tasks (values are in degrees).

RMSE	Left Ankle Angle	Right Ankle Angle	Left Knee Angle	Right Knee Angle	Left Hip Angle	Right Hip Angle
Bilateral Squat	7.52	8.04	9.31	11.57	7.55	9.22
Deadlift	5.12	5.3	7.43	8.47	13.22	13.2
Vertical Jump	9.64	10.99	12.47	10.87	8.62	9.42
Left Limb Single Leg Squat	9.45	3.3	5.99	4.83	6.09	6.99
Right Limb Single Leg Squat	4.49	8.9	6.23	8.53	5.93	6.73
Mean	7.24	7.31	8.29	8.85	8.28	9.11

**Table 2:** Valor IMU vs. Vicon RMSE in Shoulder Joint Angles. The table shows the upper extremity joint angle mean root-mean-squared-error (RMSE) observations when comparing Valor-derived joint angles to Vicon-derived joint angles across all subjects and tasks (values are in degrees). Placeholders marked by an “x” indicate data was not available.

RMSE	Left Shoulder Abduction	Right Shoulder Abduction	Left Shoulder Flexion	Right Shoulder Flexion
Left Shoulder Abduction	8.97	1.89	12.46	3.94
Right Shoulder Abduction	2.56	8.60	3.71	8.14
Deadlift	x	x	14.26	16.62
Mean	5.77	5.20	10.14	9.57

**Table 3:** Valor IMU vs. Vicon ICC3k in Lower Extremity Joint Angles. The table shows the lower extremity joint angle mean interclass correlation coefficient (ICC3k) observations with 95% confidence intervals (shown in brackets) when comparing Valor-derived joint angles to Vicon-derived joint angles across all subjects and tasks.

ICC3k	Left Ankle Angle	Right Ankle Angle	Left Knee Angle	Right Knee Angle	Left Hip Angle	Right Hip Angle
Bilateral Squat	0.77 [0.70, 0.83]	0.93 [0.90, 0.94]	0.90 [0.87, 0.92]	0.87 [0.83, 0.90]	0.90 [0.87, 0.92]	0.71 [0.62, 0.78]
Deadlift	0.89 [0.87, 0.91]	0.90 [0.88, 0.92]	0.79 [0.73, 0.83]	0.80 [0.75, 0.84]	0.89 [0.87, 0.91]	0.81 [0.76, 0.84]
Vertical Jump	0.98 [0.96, 0.98]	0.97 [0.95, 0.98]	0.93 [0.88, 0.95]	0.88 [0.81, 0.93]	0.89 [0.82, 0.93]	0.72 [0.56, 0.82]
Left Limb Single Leg Squat	0.76 [0.63, 0.85]	0.95 [0.92, 0.97]	0.94 [0.90, 0.96]	0.80 [0.67, 0.88]	0.93 [0.89, 0.95]	0.95 [0.93, 0.97]
Right Limb Single Leg Squat	0.91 [0.86, 0.94]	0.76 [0.62, 0.84]	0.85 [0.76, 0.91]	0.88 [0.81, 0.93]	0.95 [0.92, 0.97]	0.81 [0.70, 0.88]

**Table 4:** Valor IMU vs. Vicon ICC3k in Shoulder Joint Angles. The table shows the upper extremity joint angle mean interclass correlation coefficient (ICC3k) observations with 95% confidence intervals (shown in brackets) when comparing Valor-derived joint angles to Vicon-derived joint angles across all subjects and tasks. Placeholders marked by an “x” indicate data were not compared between systems.

ICC3k	Left Shoulder Abduction	Right Shoulder Abduction	Left Shoulder Flexion	Right Shoulder Flexion
Left Shoulder Abduction	0.87 [0.80, 0.92]	0.99 [0.98, 0.99]	0.11 [−0.39, 0.43]	0.30 [−0.1, 0.55]
Right Shoulder Abduction	0.98 [0.97, 0.99]	0.84 [0.75, 0.90]	x	0.06 [−0.48, 0.40]
Deadlift	x	x	0.57 [0.47, 0.65]	0.84 [0.80, 0.87]

**Table 5:** Valor IMU vs. Vicon Paired *T*-Test Results across All Joint Angles. The table shows the t-statistic and *p*-value observations for each joint angle, which were used when determining the statistical significance of the difference between Valor-derived and Vicon-derived joint angles, finding that these differences were less than 10 degrees.

Joint Angle	t-Statistic	*p*-Value (α < 0.05)
Left Ankle Angle	−24.33	7.27 × 10^−44^
Right Ankle Angle	−27.78	7.71 × 10^−49^
Left Knee Angle	−18.05	2.20 × 10^−33^
Right Knee Angle	−8.12	6.80 × 10^−13^
Left Hip Angle	−8.49	3.19 × 10^−13^
Right Hip Angle	−7.22	1.09 × 10^−10^
Left Shoulder Abduction	−6.35	8.45 × 10^−8^
Right Shoulder Abduction	−7.28	4.42 × 10^−9^
Left Shoulder Flexion	−10.21	5.79 × 10^−15^
Right Shoulder Flexion	−10.26	4.83 × 10^−15^

## Data Availability

The original contributions presented in the study are included in the article/Supplementary Material, further inquiries can be directed to the corresponding author.

## Supplementary Materials

The following supporting information can be downloaded at: https://www.mdpi.com/article/10.3390/s24175833/s1.
