# Supplementary material for: Validity of Valor Inertial Measurement Unit for Upper and Lower Extremity Joint Angles
Source: Sensors (Basel). 2024 Sep 8;24(17):5833. doi: 10.3390/s24175833 (PMC11397965; doi:10.3390/s24175833)
Supplement: Supplementary file 1 [file sensors-24-05833-s001.zip › sensors-3126928-supplementary.pdf]

Mean absolute differences between Valor IMU and Vicon motion capture across all subjects' joint angles and tasks ranged from 1.81 degrees to 17.46 degrees (**Tables S1 & S2**). In the lower extremity, the lowest reported mean absolute difference was 2.88 degrees in the right ankle during left limb single leg squat while the highest reported mean absolute difference was 14.66 degrees in the right hip during deadlifting (**Table S1**). Regarding mean absolute difference in the shoulder joint angles, the lowest observed value was 1.81 degrees during right shoulder abduction while the highest mean absolute difference of 17.45 degrees was found in right shoulder flexion (**Table S1**). Our results reveal the commercially available Valor IMU suit produced similar joint angles when compared to Vicon optical motion capture.

Table S1. Valor IMU vs Vicon Mean Absolute Difference in Lower Extremity Joint Angle. Results of mean absolute difference in lower extremity joint angles in degrees. All subjects and their respective joint angles across movements when comparing the Valor inertia measurement unit to Vicon motion capture are represented by the average and 95% confidence intervals (shown in brackets).

**Mean Absolute Difference**	**Left Ankle Angle**	**Right Ankle Angle**	**Left Knee Angle**	**Right Knee Angle**	**Left Hip Angle**	**Right Hip Angle**
Bilateral Squat	7.02 [6.58, 7.45]	7.69 [7.27, 8.10]	8.16 [7.08, 9.25]	9.28 [8.19,10.37]	7.29 [6.46, 8.13]	8.27 [7.25, 9.28]
Deadlift	4.30 [4.03, 4.57]	4.69 [4.41, 4.98]	7.17 [6.50, 7.85]	8.98 [8.28, 9.67]	14.13 [13.26, 15.00]	14.66 [13.73, 15.59]
Vertical Jump	9.00 [7.91, 10.09]	10.42 [9.21, 11.64]	11.92 [10.35, 13.49]	10.04 [8.24, 11.85]	7.93 [6.55, 9.31]	8.87 [7.39, 10.36]
Left Limb Single Leg Squat	9.30 [8.32, 10.28]	2.88 [2.22, 3.54]	5.83 [4.72, 6.94]	4.46 [3.69, 5.22]	5.98 [4.70, 7.27]	6.86 [5.54, 8.19]
Right Limb Single Leg Squat	3.57 [2.38, 4.75]	8.71 [7.59, 9.82]	5.21 [3.93, 6.48]	8.33 [6.60, 10.06]	5.79 [4.48, 7.11]	6.69 [5.17, 8.21]

Table S2. Valor IMU vs Vicon Mean Absolute Difference in Shoulder Joint Angle. Results of mean absolute difference in upper extremity joint angles in degrees. All subjects and their respective joint angles across movements when comparing the Valor inertia measurement unit to Vicon motion capture are represented by the average and 95% confidence intervals (shown in brackets). Placeholders marked by an "x" indicate data was not available. Data that is not applicable is represented by "N/A".

**Mean Absolute Difference**	**Left Shoulder Abduction**	**Right Shoulder Abduction**	**Left Shoulder Flexion**	**Right Shoulder Flexion**
Left Shoulder Abduction	8.52 [6.96, 10.08]	N/A	12.18 [10.30, 14.05]	3.94 [3.25, 4.62]
Right Shoulder Abduction	N/A	8.40 [7.04, 9.76]	3.46 [2.81, 4.11]	7.99 [6.47, 9.52]
Deadlift	x	x	12.16 [11.13, 13.18]	17.46 [16.65, 18.26]
